# (*Z*)-3-*p*-Tolyl-2-(*p*-tolyl­imino)-1,3-thia­zolidin-4-one

**DOI:** 10.1107/S160053681201149X

**Published:** 2012-03-24

**Authors:** Hatem A. Abdel-Aziz, Hazem A. Ghabbour, Tze Shyang Chia, Hoong-Kun Fun

**Affiliations:** a Department of Pharmaceutical Chemistry, College of Pharmacy, King Saud University, PO Box 2457, Riyadh 11451, Saudi Arabia; b X-ray Crystallography Unit, School of Physics, Universiti Sains Malaysia, 11800 USM, Penang, Malaysia

## Abstract

In the title compound, C_17_H_16_N_2_OS, the central thia­zolidin-4-one ring forms dihedral angles of 66.49 (9) and 79.45 (6)° with the two methyl-substituted benzene rings. In the crystal, mol­ecules are stacked in columns along the *b* axis through C—H⋯π inter­actions. The H atoms of one of the methyl groups are disordered over two orientations with equal site occupancies.

## Related literature

For the chemistry of thia­zolidin-4-one and its experimental preparation, see: Abdel-Aziz *et al.* (2010 ▶). For a related structure, see: Zeller *et al.* (2011 ▶). For reference bond lengths, see: Allen *et al.* (1987 ▶).
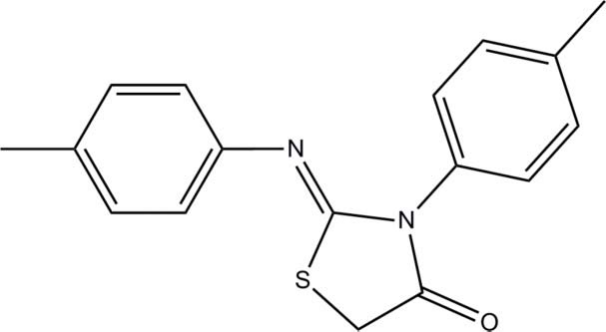


## Experimental

### 

#### Crystal data

C_17_H_16_N_2_OS
*M* *_r_* = 296.38
Monoclinic, 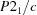

*a* = 14.1321 (4) Å
*b* = 5.8524 (2) Å
*c* = 19.0076 (6) Å
β = 100.307 (2)°
*V* = 1546.69 (8) Å^3^
*Z* = 4
Cu *K*α radiation
μ = 1.85 mm^−1^
*T* = 296 K
0.98 × 0.21 × 0.06 mm

#### Data collection

Bruker SMART APEXII CCD area-detector diffractometer
Absorption correction: multi-scan (*SADABS*; Bruker, 2009 ▶) *T*_min_ = 0.264, *T*_max_ = 0.897
10830 measured reflections
2849 independent reflections
2293 reflections with *I* > 2σ(*I*)
*R*_int_ = 0.041

#### Refinement

*R*[*F* ^2^ > 2σ(*F* ^2^)] = 0.046
*wR*(*F* ^2^) = 0.135
*S* = 1.07
2849 reflections
194 parameters
H-atom parameters constrained
Δρ_max_ = 0.35 e Å^−3^
Δρ_min_ = −0.35 e Å^−3^

### 

Data collection: *APEX2* (Bruker, 2009 ▶); cell refinement: *SAINT* (Bruker, 2009 ▶); data reduction: *SAINT*; program(s) used to solve structure: *SHELXTL* (Sheldrick, 2008 ▶); program(s) used to refine structure: *SHELXTL*; molecular graphics: *SHELXTL*; software used to prepare material for publication: *SHELXTL* and *PLATON* (Spek, 2009 ▶).

## Supplementary Material

Crystal structure: contains datablock(s) global, I. DOI: 10.1107/S160053681201149X/is5090sup1.cif

Structure factors: contains datablock(s) I. DOI: 10.1107/S160053681201149X/is5090Isup2.hkl

Supplementary material file. DOI: 10.1107/S160053681201149X/is5090Isup3.cml

Additional supplementary materials:  crystallographic information; 3D view; checkCIF report

## Figures and Tables

**Table 1:** Hydrogen-bond geometry (Å, °) *Cg*1 and *Cg*2 are the centroids of the S1/N2/C1–C3 and C4–C9 rings, respectively.

*D*—H⋯*A*	*D*—H	H⋯*A*	*D*⋯*A*	*D*—H⋯*A*
C5—H5*A*⋯*Cg*1^i^	0.93	3.00	3.788 (2)	144
C9—H9*A*⋯*Cg*2^ii^	0.93	2.87	3.607 (2)	138

Symmetry codes: (i) 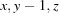; (ii) 
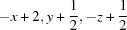
.

**Fig. 1.**
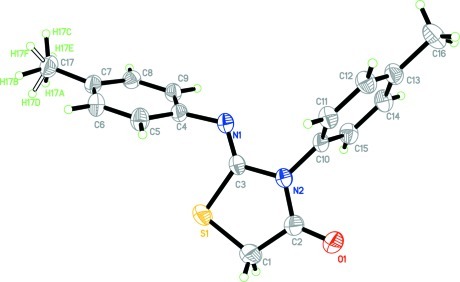
The molecular structure of the title compound with atom labels and 30% probability displacement ellipsoids.

## supplementary crystallographic information

### Comment

The molecular structure of the title compound is shown in Fig. 1. The mean planes of the two methyl-substituted benzene rings (C4–C9 & C10–C15) make dihedral angles of 66.49 (9) and 79.45 (6)°, respectively, with the mean plane of the central thiazolidin-4-one ring [S1/N2/C1–C3/O1; maximum deviation = 0.0075 (12) Å at atom C3]. In the molecule, the hydrogen atoms which are attached to atom C17 are disordered over two positions, with equal site-occupancies. Bond lengths (Allen *et al.*, 1987) and angles are within normal ranges and are comparable to a related structure (Zeller *et al.*, 2011).

In the crystal structure, no significant intermolecular hydrogen bonds are observed. The crystal structure is stabilized by C—H···π interactions (Table 1), involving *Cg*1 and *Cg*2 which are the centroids of S1/N2/C1–C3 and C4–C9 rings, respectively.

### Experimental

The title compound was prepared according to the reported method by Abdel-Aziz *et al.* (2010). Crystals of the title compound were obtained by slow evaporation from an ethanol solution at room temperature.

### Refinement

The methyl group with atom C17 was found to be disordered over two orientations and the H atoms were located in a difference Fourier map. The site-occupancy ratio was refined to 0.52 (3):0.48 (3) in the refinement using C—H bond distance restraints. In the final refinement, the occupancies were fixed at 0.5 and the H atoms were treated as riding (C—H = 0.96 Å), with *U*_iso_(H) = 1.5*U*_eq_(C). A rotating group model was used for each of the disordered components. All other H atoms were positioned geometrically (C—H = 0.93, 0.96 or 0.97 Å) and refined using a riding model with *U*_iso_(H) = 1.2 or 1.5*U*_eq_(C). A rotating group model was also applied to the other methyl group.

### Crystal data

**Table:**

C_17_H_16_N_2_OS	*F*(000) = 624
*M**_r_* = 296.38	*D*_x_ = 1.273 Mg m^−^^3^
Monoclinic, *P*2_1_/*c*	Cu *K*α radiation, λ = 1.54178 Å
Hall symbol: -P 2ybc	Cell parameters from 1135 reflections
*a* = 14.1321 (4) Å	θ = 4.7–69.2°
*b* = 5.8524 (2) Å	µ = 1.85 mm^−^^1^
*c* = 19.0076 (6) Å	*T* = 296 K
β = 100.307 (2)°	Needle, colourless
*V* = 1546.69 (8) Å^3^	0.98 × 0.21 × 0.06 mm
*Z* = 4	

### Data collection

**Table:**

Bruker SMART APEXII CCD area-detector diffractometer	2849 independent reflections
Radiation source: fine-focus sealed tube	2293 reflections with *I* > 2σ(*I*)
Graphite monochromator	*R*_int_ = 0.041
φ and ω scans	θ_max_ = 69.8°, θ_min_ = 4.7°
Absorption correction: multi-scan (*SADABS*; Bruker, 2009)	*h* = −16→17
*T*_min_ = 0.264, *T*_max_ = 0.897	*k* = −5→6
10830 measured reflections	*l* = −23→22

### Refinement

**Table:**

Refinement on *F*^2^	Secondary atom site location: difference Fourier map
Least-squares matrix: full	Hydrogen site location: inferred from neighbouring sites
*R*[*F*^2^ > 2σ(*F*^2^)] = 0.046	H-atom parameters constrained
*wR*(*F*^2^) = 0.135	*w* = 1/[σ^2^(*F*_o_^2^) + (0.0633*P*)^2^ + 0.2896*P*] where *P* = (*F*_o_^2^ + 2*F*_c_^2^)/3
*S* = 1.07	(Δ/σ)_max_ = 0.001
2849 reflections	Δρ_max_ = 0.35 e Å^−^^3^
194 parameters	Δρ_min_ = −0.35 e Å^−^^3^
0 restraints	Extinction correction: *SHELXTL* (Sheldrick, 2008), Fc^*^=kFc[1+0.001xFc^2^λ^3^/sin(2θ)]^-1/4^
Primary atom site location: structure-invariant direct methods	Extinction coefficient: 0.0124 (10)

### Special details

**Table:**

Geometry. All e.s.d.'s (except the e.s.d. in the dihedral angle between two l.s. planes) are estimated using the full covariance matrix. The cell e.s.d.'s are taken into account individually in the estimation of e.s.d.'s in distances, angles and torsion angles; correlations between e.s.d.'s in cell parameters are only used when they are defined by crystal symmetry. An approximate (isotropic) treatment of cell e.s.d.'s is used for estimating e.s.d.'s involving l.s. planes.
Refinement. Refinement of *F*^2^ against ALL reflections. The weighted *R*-factor *wR* and goodness of fit *S* are based on *F*^2^, conventional *R*-factors *R* are based on *F*, with *F* set to zero for negative *F*^2^. The threshold expression of *F*^2^ > σ(*F*^2^) is used only for calculating *R*-factors(gt) *etc*. and is not relevant to the choice of reflections for refinement. *R*-factors based on *F*^2^ are statistically about twice as large as those based on *F*, and *R*- factors based on ALL data will be even larger.

### Fractional atomic coordinates and isotropic or equivalent isotropic displacement parameters (Å^2^)

**Table:**

	*x*	*y*	*z*	*U*_iso_*/*U*_eq_	Occ. (<1)
S1	0.86258 (4)	−0.00010 (12)	0.47164 (3)	0.0765 (3)	
N1	0.84789 (10)	−0.0771 (3)	0.32906 (8)	0.0522 (4)	
N2	0.73053 (10)	0.1441 (3)	0.36745 (8)	0.0523 (4)	
O1	0.63384 (14)	0.3619 (4)	0.42357 (10)	0.1118 (8)	
C1	0.7698 (2)	0.1807 (7)	0.49367 (13)	0.1031 (11)	
H1A	0.7349	0.1019	0.5260	0.124*	
H1B	0.7977	0.3185	0.5171	0.124*	
C2	0.70278 (16)	0.2406 (5)	0.42561 (12)	0.0757 (7)	
C3	0.81438 (12)	0.0103 (3)	0.37930 (10)	0.0496 (4)	
C4	0.93412 (12)	−0.2091 (3)	0.34399 (9)	0.0479 (4)	
C5	0.93843 (14)	−0.4167 (3)	0.37968 (12)	0.0584 (5)	
H5A	0.8857	−0.4678	0.3983	0.070*	
C6	1.02137 (14)	−0.5478 (3)	0.38750 (12)	0.0591 (5)	
H6A	1.0236	−0.6864	0.4117	0.071*	
C7	1.10064 (13)	−0.4772 (3)	0.36010 (11)	0.0515 (5)	
C8	1.09587 (13)	−0.2684 (3)	0.32587 (10)	0.0530 (4)	
H8A	1.1492	−0.2155	0.3083	0.064*	
C9	1.01362 (13)	−0.1362 (3)	0.31707 (10)	0.0514 (4)	
H9A	1.0117	0.0025	0.2929	0.062*	
C10	0.67884 (12)	0.1860 (3)	0.29574 (10)	0.0475 (4)	
C11	0.61531 (13)	0.0238 (3)	0.26253 (11)	0.0535 (5)	
H11A	0.6064	−0.1124	0.2857	0.064*	
C12	0.56500 (14)	0.0659 (4)	0.19452 (11)	0.0584 (5)	
H12A	0.5222	−0.0434	0.1721	0.070*	
C13	0.57711 (13)	0.2673 (4)	0.15916 (10)	0.0561 (5)	
C14	0.64077 (14)	0.4275 (4)	0.19421 (11)	0.0592 (5)	
H14A	0.6495	0.5644	0.1714	0.071*	
C15	0.69157 (13)	0.3888 (3)	0.26226 (11)	0.0563 (5)	
H15A	0.7338	0.4986	0.2851	0.068*	
C16	0.52221 (18)	0.3121 (5)	0.08465 (13)	0.0838 (8)	
H16A	0.4567	0.2639	0.0816	0.126*	
H16B	0.5512	0.2284	0.0506	0.126*	
H16C	0.5239	0.4725	0.0744	0.126*	
C17	1.18878 (15)	−0.6264 (4)	0.36447 (13)	0.0688 (6)	
H17A	1.2442	−0.5449	0.3885	0.103*	0.50
H17B	1.1977	−0.6659	0.3171	0.103*	0.50
H17C	1.1804	−0.7631	0.3906	0.103*	0.50
H17D	1.2256	−0.6203	0.4120	0.103*	0.50
H17E	1.2273	−0.5724	0.3311	0.103*	0.50
H17F	1.1694	−0.7811	0.3530	0.103*	0.50

### Atomic displacement parameters (Å^2^)

**Table:**

	*U*^11^	*U*^22^	*U*^33^	*U*^12^	*U*^13^	*U*^23^
S1	0.0617 (4)	0.1143 (5)	0.0480 (4)	0.0285 (3)	−0.0047 (2)	0.0048 (3)
N1	0.0461 (8)	0.0559 (9)	0.0516 (9)	0.0101 (7)	0.0007 (6)	0.0030 (7)
N2	0.0441 (8)	0.0635 (10)	0.0461 (8)	0.0110 (7)	−0.0010 (6)	−0.0011 (7)
O1	0.0957 (13)	0.165 (2)	0.0691 (11)	0.0751 (14)	−0.0007 (9)	−0.0207 (12)
C1	0.0900 (18)	0.159 (3)	0.0529 (13)	0.0537 (19)	−0.0063 (12)	−0.0151 (16)
C2	0.0616 (12)	0.1040 (18)	0.0575 (12)	0.0279 (13)	−0.0003 (10)	−0.0105 (12)
C3	0.0412 (9)	0.0541 (10)	0.0502 (10)	0.0044 (7)	−0.0009 (8)	0.0044 (8)
C4	0.0464 (9)	0.0470 (9)	0.0472 (9)	0.0061 (7)	0.0003 (7)	−0.0010 (7)
C5	0.0487 (10)	0.0565 (11)	0.0701 (13)	0.0018 (8)	0.0110 (9)	0.0098 (9)
C6	0.0561 (11)	0.0474 (11)	0.0718 (13)	0.0066 (8)	0.0062 (9)	0.0119 (9)
C7	0.0491 (10)	0.0494 (10)	0.0530 (10)	0.0064 (7)	0.0013 (8)	−0.0053 (8)
C8	0.0492 (9)	0.0546 (11)	0.0550 (10)	0.0001 (8)	0.0092 (8)	−0.0027 (8)
C9	0.0559 (10)	0.0455 (10)	0.0516 (10)	0.0038 (8)	0.0061 (8)	0.0039 (7)
C10	0.0377 (8)	0.0539 (10)	0.0477 (9)	0.0077 (7)	−0.0010 (7)	−0.0008 (7)
C11	0.0495 (10)	0.0507 (10)	0.0573 (11)	−0.0010 (7)	0.0017 (8)	0.0047 (8)
C12	0.0487 (10)	0.0642 (12)	0.0575 (11)	−0.0066 (8)	−0.0030 (8)	−0.0023 (9)
C13	0.0456 (9)	0.0677 (12)	0.0524 (11)	0.0038 (8)	0.0017 (8)	0.0036 (9)
C14	0.0572 (11)	0.0557 (11)	0.0625 (12)	0.0008 (9)	0.0053 (9)	0.0117 (9)
C15	0.0492 (10)	0.0516 (11)	0.0639 (12)	−0.0032 (8)	−0.0011 (8)	−0.0037 (9)
C16	0.0790 (15)	0.108 (2)	0.0574 (13)	−0.0013 (14)	−0.0077 (11)	0.0135 (13)
C17	0.0595 (11)	0.0652 (13)	0.0810 (15)	0.0165 (10)	0.0111 (10)	0.0006 (11)

### Geometric parameters (Å, º)

**Table:**

S1—C3	1.7664 (19)	C9—H9A	0.9300
S1—C1	1.792 (3)	C10—C15	1.374 (3)
N1—C3	1.249 (2)	C10—C11	1.379 (3)
N1—C4	1.428 (2)	C11—C12	1.381 (3)
N2—C2	1.360 (3)	C11—H11A	0.9300
N2—C3	1.405 (2)	C12—C13	1.383 (3)
N2—C10	1.448 (2)	C12—H12A	0.9300
O1—C2	1.200 (3)	C13—C14	1.385 (3)
C1—C2	1.502 (3)	C13—C16	1.511 (3)
C1—H1A	0.9700	C14—C15	1.381 (3)
C1—H1B	0.9700	C14—H14A	0.9300
C4—C9	1.383 (3)	C15—H15A	0.9300
C4—C5	1.387 (3)	C16—H16A	0.9600
C5—C6	1.387 (3)	C16—H16B	0.9600
C5—H5A	0.9300	C16—H16C	0.9600
C6—C7	1.381 (3)	C17—H17A	0.9600
C6—H6A	0.9300	C17—H17B	0.9600
C7—C8	1.380 (3)	C17—H17C	0.9600
C7—C17	1.511 (3)	C17—H17D	0.9600
C8—C9	1.381 (3)	C17—H17E	0.9600
C8—H8A	0.9300	C17—H17F	0.9600
			
C3—S1—C1	92.50 (10)	C4—C9—H9A	119.9
C3—N1—C4	119.78 (15)	C15—C10—C11	120.72 (17)
C2—N2—C3	117.32 (15)	C15—C10—N2	119.79 (16)
C2—N2—C10	121.64 (15)	C11—C10—N2	119.46 (17)
C3—N2—C10	120.97 (15)	C10—C11—C12	119.31 (18)
C2—C1—S1	108.16 (17)	C10—C11—H11A	120.3
C2—C1—H1A	110.1	C12—C11—H11A	120.3
S1—C1—H1A	110.1	C11—C12—C13	121.27 (18)
C2—C1—H1B	110.1	C11—C12—H12A	119.4
S1—C1—H1B	110.1	C13—C12—H12A	119.4
H1A—C1—H1B	108.4	C12—C13—C14	118.00 (17)
O1—C2—N2	124.7 (2)	C12—C13—C16	121.1 (2)
O1—C2—C1	123.3 (2)	C14—C13—C16	120.9 (2)
N2—C2—C1	111.96 (18)	C15—C14—C13	121.58 (19)
N1—C3—N2	122.02 (16)	C15—C14—H14A	119.2
N1—C3—S1	127.89 (14)	C13—C14—H14A	119.2
N2—C3—S1	110.05 (13)	C10—C15—C14	119.10 (17)
C9—C4—C5	118.94 (17)	C10—C15—H15A	120.4
C9—C4—N1	118.71 (16)	C14—C15—H15A	120.4
C5—C4—N1	122.16 (17)	C13—C16—H16A	109.5
C6—C5—C4	119.96 (19)	C13—C16—H16B	109.5
C6—C5—H5A	120.0	H16A—C16—H16B	109.5
C4—C5—H5A	120.0	C13—C16—H16C	109.5
C7—C6—C5	121.40 (18)	H16A—C16—H16C	109.5
C7—C6—H6A	119.3	H16B—C16—H16C	109.5
C5—C6—H6A	119.3	C7—C17—H17A	109.5
C8—C7—C6	117.93 (17)	C7—C17—H17B	109.5
C8—C7—C17	120.51 (19)	C7—C17—H17C	109.5
C6—C7—C17	121.51 (19)	C7—C17—H17D	109.5
C7—C8—C9	121.47 (18)	C7—C17—H17E	109.5
C7—C8—H8A	119.3	H17D—C17—H17E	109.5
C9—C8—H8A	119.3	C7—C17—H17F	109.5
C8—C9—C4	120.27 (18)	H17D—C17—H17F	109.5
C8—C9—H9A	119.9	H17E—C17—H17F	109.5
			
C3—S1—C1—C2	0.6 (3)	C5—C6—C7—C17	−176.1 (2)
C3—N2—C2—O1	178.7 (3)	C6—C7—C8—C9	−2.0 (3)
C10—N2—C2—O1	1.7 (4)	C17—C7—C8—C9	175.62 (19)
C3—N2—C2—C1	−0.4 (3)	C7—C8—C9—C4	1.3 (3)
C10—N2—C2—C1	−177.4 (2)	C5—C4—C9—C8	−0.1 (3)
S1—C1—C2—O1	−179.3 (3)	N1—C4—C9—C8	−175.14 (17)
S1—C1—C2—N2	−0.2 (4)	C2—N2—C10—C15	77.3 (3)
C4—N1—C3—N2	179.14 (16)	C3—N2—C10—C15	−99.6 (2)
C4—N1—C3—S1	1.9 (3)	C2—N2—C10—C11	−101.0 (2)
C2—N2—C3—N1	−176.8 (2)	C3—N2—C10—C11	82.1 (2)
C10—N2—C3—N1	0.2 (3)	C15—C10—C11—C12	0.7 (3)
C2—N2—C3—S1	0.9 (2)	N2—C10—C11—C12	179.01 (17)
C10—N2—C3—S1	177.91 (14)	C10—C11—C12—C13	0.0 (3)
C1—S1—C3—N1	176.7 (2)	C11—C12—C13—C14	−0.5 (3)
C1—S1—C3—N2	−0.82 (19)	C11—C12—C13—C16	179.7 (2)
C3—N1—C4—C9	−118.4 (2)	C12—C13—C14—C15	0.4 (3)
C3—N1—C4—C5	66.7 (3)	C16—C13—C14—C15	−179.9 (2)
C9—C4—C5—C6	−0.4 (3)	C11—C10—C15—C14	−0.9 (3)
N1—C4—C5—C6	174.46 (19)	N2—C10—C15—C14	−179.14 (17)
C4—C5—C6—C7	−0.3 (3)	C13—C14—C15—C10	0.3 (3)
C5—C6—C7—C8	1.5 (3)		

### Hydrogen-bond geometry (Å, º)

*Cg*1 and *Cg*2 are the centroids of the S1/N2/C1–C3 and C4–C9 rings, respectively.

**Table:**

*D*—H···*A*	*D*—H	H···*A*	*D*···*A*	*D*—H···*A*
C5—H5*A*···*Cg*1^i^	0.93	3.00	3.788 (2)	144
C9—H9*A*···*Cg*2^ii^	0.93	2.87	3.607 (2)	138

Symmetry codes: (i) *x*, *y*−1, *z*; (ii) −*x*+2, *y*+1/2, −*z*+1/2.
